# Protocol for an Exploratory RCT of the Traumatic Stress Relief Intervention With Persons With Lived Experience of Leprosy in Addis Ababa, Ethiopia

**DOI:** 10.1155/jotm/1307578

**Published:** 2025-03-13

**Authors:** Safa Kemal Kaptan, Marekegn Habtamu, Solomon Getahun, Beletshachew Tadesse, Ayse Akan, Adeline Pupat, Cécile Bizouerne, Nusrat Husain

**Affiliations:** ^1^Department of Psychology, Boğaziçi University, İstanbul, Türkiye; ^2^The Leprosy Mission International Ethiopia, Addis Ababa, Ethiopia; ^3^Private Practice, Toulouse, France; ^4^Private Practice, Paris, France; ^5^Division of Psychology and Mental Health, School of Health Sciences, University of Manchester, Manchester, UK

**Keywords:** Ethiopia, intervention, leprosy, mental health, neglected tropical diseases, PTSD, trauma, Traumatic Stress Relief

## Abstract

**Objectives:** Neglected tropical diseases (NTDs), such as leprosy, significantly impact mental health and overall well-being. This study aims to evaluate the effectiveness and acceptability of a mental health intervention targeting individuals affected by leprosy in Ethiopia. The intervention utilizes the Traumatic Stress Relief (TSR) program to target symptoms of posttraumatic stress disorder (PTSD), anxiety, and depression, and to improve overall mental health.

**Methods:** This exploratory randomized controlled trial (RCT) will recruit participants with lived experiences of leprosy. Participants will receive four group sessions of a low-intensity TSR intervention. The intervention will be administered by a pair of trained facilitators, including one mental health practitioner and one community member with lived experience of leprosy. Data will be collected through self-report questionnaires to assess changes in PTSD symptoms, anxiety, and depression. In addition, interviews will provide further insights into participants' experiences and the acceptability of the intervention.

**Discussion:** This exploratory trial will provide insights into the feasibility of mental health interventions for individuals affected by leprosy. The findings will inform the design of future trials to evaluate the effectiveness of such programs on a larger scale and in more diverse contexts.

**Trial Registration:** The UK's Clinical Study Registry: ISRCTN868254411

**Summary**

• The Traumatic Stress Relief (TSR) program addresses a critical gap in mental health support for individuals affected by leprosy, a population often marginalized and overlooked in healthcare systems.
• By implementing a peer-supported group therapy model in Addis Ababa, Ethiopia, this research provides a scalable framework for improving psychological well-being among leprosy patients.
• The program not only targets symptoms of posttraumatic stress disorder (PTSD), anxiety, and depression but also aims to reduce stigma and enhance social support networks within the community.
• The impact of this research extends beyond immediate health benefits; it has the potential to inform public health policies and practices at local, national, and international levels.
• By demonstrating the effectiveness of community-based mental health interventions, the TSR program can influence policymakers to adopt similar approaches for other neglected diseases and vulnerable populations.
• Finally, the findings of this study may inspire further peer-supported strategies, fostering a more integrated approach to healthcare that recognizes the importance of mental health alongside physical health.

## 1. Introduction

Neglected tropical diseases (NTDs) are widespread in many developing tropical countries, leading to significant illness and impeding economic development in communities [1]. The research indicates that individuals with NTDs often experience a reduced quality of life due to their symptoms, emotional distress, and treatment challenges [2]. Specifically, those with visible deformities from conditions such as leprosy face significant difficulties [3].

Leprosy is a condition that primarily affects the skin and peripheral nerves. Leprosy remains a significant public health concern in many parts of the world with more than 200,000 new cases reported annually [4, 5]. Although leprosy is treatable and early intervention can prevent disability, the visible impairments and the stigma associated with the disease lead to challenges such as social exclusion [6], income loss and unemployment, and problems in access to public services in health and education [7], which all have been associated with poor well-being [8, 9]. In their study on mental health difficulties with leprosy patients, Soni, Kaur, and Kaur [10] reported that 14% of the participants had moderate to severe depression and almost one in five patients who showed symptoms of depression had attempted suicide. In another study, the overall prevalence of mental health difficulties such as depression and anxiety was found to be 34.6% among persons affected by leprosy [11]. Further studies from other leprosy endemic countries also reported a high prevalence of mental health difficulties [12–14].

However, the research on leprosy has primarily focused on its physical aspects, despite recent studies highlighting the need for mental health interventions to address the trauma caused by the disease [8]. Recent studies emphasize that while existing evidence supports increased investment in mental health interventions for individuals affected by NTDs, including leprosy, there remains a significant need for research [4, 15]. Thus, recent attention has begun to focus on health-seeking behaviors and recovery, although psychosocial interventions targeting leprosy-related well-being remain scarce due to limited resources in endemic countries, with a few exceptions [15, 16].

Further to that, low-resource settings, including many low- and middle-income countries (LMICs) and disaster-stricken regions, face severe shortages of mental health professionals [17]. For example, there are only 2.8 mental health workers per 100,000 people in South-East Asia and 1.6 in Africa, compared to 44.8 in Europe [18]. In Sierra Leone, for instance, there were just two psychiatrists for six million people following the Ebola outbreak, and in Ethiopia, there is about one psychiatrist per two million people and only an estimated 0.68 mental health professionals per 100,000 people.

In response, the World Health Organization (WHO) advocates for the use of scalable psychological interventions. These interventions are brief, manualized, and relatively easy to follow, making them suitable for implementation by trained and supervised nonspecialist staff. Designed to be scalable and adaptable, such interventions utilize new technologies or group formats to enhance accessibility. They can be deployed in various settings, including refugee camps, disaster zones, hospitals, and schools, effectively addressing common mental health disorders and often at no cost.

One example of scalable intervention, TSR, is the flagship program of Global Initiative for Stress and Trauma Treatment (GIST-T) developed by an international team of 25 experts from 13 countries. The program has been designed to train frontline professionals and nonspecialist staff to provide relief, especially in LMICs, by providing a structured, evidence-based approach to reduce symptoms of mental health difficulties [19].

The current project will test TSR's potential effectiveness in reducing symptoms of PTSD, anxiety, and depression. Specifically, it will evaluate the feasibility of recruiting individuals with leprosy for the TSR intervention, assessing its acceptability through various parameters such as recruitment rates, participant engagement, session attendance, and dropout rates. Qualitative interviews will further explore these aspects. The study will also define appropriate primary and secondary outcomes for assessing the intervention's effect in a full-scale trial. Data from this study will inform the design of a future multisite definitive clinical trial, including optimizing the design and planning the sample size. By focusing on this underserved population, the study aims to fill a critical gap in the literature and provide a foundation for future research on the effectiveness of mental health interventions for individuals with leprosy. The findings will have significant implications for public health strategies in similar contexts and could lead to the development of more comprehensive care models that address both the physical and psychological needs of individuals affected by leprosy.

## 2. Methods

The SPIRIT 2013 Statement: Defining standard protocol items for clinical trials was used in reporting this protocol. The trials received ethical approval from the City Government of Addis Ababa Health Bureau.

### 2.1. Study Design

This randomized controlled trial (RCT) with a qualitative component is designed to assess the effectiveness of the TSR intervention for individuals affected by leprosy in Addis Ababa, Ethiopia.

Methodological features of this feasibility RCT will minimize the possible bias in the intervention group. These features include allocation concealment (that will be ensured by randomization to be performed by an independent, off-site statistician), blinding (study statistician and the research team carrying out follow-up assessments will be masked to treatment allocation), use of intention-to-treat analysis, and the use of primary versus secondary endpoints.

The study will involve two arms: an intervention group receiving the TSR intervention and a waitlist control group. Participants will undergo individual randomization conducted by an off-site statistician. A total of 100 participants will be randomized in a 1:1 ratio into either the TSR intervention group (*n* = 50) or the waitlist control group (*n* = 50). Randomization will occur after baseline assessments using a computer-generated sequence to ensure allocation concealment.

As this study first of its kind and aims to demonstrate a trend in favor of the intervention and the recruitment and retention of participants into the study, the sample size was informed by community consultation and trial guidelines [20–25] to ensure a sample that allows for the estimation of recruitment and retention parameters, as well as potential effectiveness.

Participants will be included in the study if they meet the following criteria: (a) aged over 18 years, (b) able to give written informed consent—individuals with physical disabilities that may limit their ability to provide written consent will be permitted to give verbal consent, (c) leprosy-affected persons residing in the research sites, (d) score positive for depression on the Patient Health Questionnaire (PHQ-9), and (e) registered with a local health service. Although, to the best of our knowledge, there is no systematic mental health support specifically designed for individuals affected by leprosy, the limited available care may include counseling, therapeutic interventions, and pharmacological treatment, based on the severity of the condition. Such services are predominantly accessible within hospitals. However, these resources are frequently inadequate in rural regions, where both clinical expertise and diagnostic capabilities are often lacking. Consequently, individuals currently receiving any form of mental health support, such as individual or group psychotherapy, will be excluded from the study, as such interventions may confound the outcomes. Furthermore, individuals presenting with dissociation, psychosis, suicidal ideation, or substance use disorders will be deemed ineligible for participation. All participants will receive compensation for their time and contribution on a per-session basis.

Participants will be recruited from community settings where persons affected by leprosy reside, in partnership with organizations of persons affected by leprosy, with whom The Leprosy Mission Ethiopia have a long-standing working relationship such as ALERT Hospital and Ethiopian National Association of Persons Affected by Leprosy (ENAPAL).

Participants will also be recruited via referrals, and announcements will be shared with relevant organizations. Stakeholder engagement exercises will be conducted. Snowball sampling will be employed to initiate contact and encourage trial participation.

Eligible participants expressing interest in further information will be contacted by a member of the research team and provided with information about the study. This will include the aims of the study and a participant information sheet.

Participants will have up to 48 h to consider their participation in the study. Upon expressing interest, they will be provided with sufficient time to review and complete a consent form, followed by the assessment tools. The assessments will be administered by trained members of the research team at the participant's preferred location and conducted in their preferred language. While most assessments are self-report measures, the research team will be available to provide assistance to participants who prefer an interview-style format. This is particularly important for questions that may be complex to interpret or could elicit emotional responses. In addition, the research team will have access to support from mental health professionals at ALERT Hospital and the ENAPAL, if needed, during the screening process.

### 2.2. Intervention

Once consent is received, participants will attend four group sessions, delivered over a period of approximately three weeks. These sessions will be conducted in person at a designated location in Addis Ababa, with each group consisting of six to eight participants. Each session will last approximately 60–90 min and will focus on addressing the psychological and social impacts of leprosy. The sessions are designed to reduce symptoms of PTSD, anxiety, and depression. Please see Table 1 for further details.

The sessions will be facilitated by a pair of one mental health professional and one peer with lived experience of leprosy. While the primary facilitator delivers the session content, the peer facilitator will provide support based on their lived experience, creating a relatable and supportive environment. All sessions will also be monitored by a senior local therapist to ensure the highest standards of care and to provide immediate additional support if needed. Furthermore, all facilitators will follow a detailed protocol and a fidelity checklist and will receive ongoing supervision from experienced trainers to ensure that the intervention is delivered consistently and effectively. These trainers have over 25 years of experience in delivering protocols for individuals with mental health difficulties and psychosocial distress, as well as in training interventions in LMICs, with 8 years specifically focused on TSR.

The TSR training has many strategies [26, 27] to improve the accessibility and acceptability which is critical as persons affected by leprosy show low use of mental health services [28, 29]. For example, it does not require participants to share traumatic experiences with others and uses activities to reduce stigma [30, 31]. It can also be framed as both a training and a mental health intervention and delivered by local providers to beneficiaries. As a result, TSR content and teaching techniques have already been found feasible in Uganda, Rwanda, and Democratic Republic of Congo [19].

Furthermore, the intervention manual and all related materials have been reviewed by a group of representatives from the ENAPAL, The Leprosy Mission International Ethiopia, and mental health professionals to ensure cultural sensitivity.

### 2.3. Data Collection

#### 2.3.1. Outcomes

The primary outcomes will be the changes in PTSD, anxiety, and depression symptoms, and levels of social support from baseline to postintervention. Secondary outcomes will include participant satisfaction and insights derived from the qualitative data.

Participants will complete a battery of self-report questionnaires at three time points: baseline (T0), postintervention (T1), and at a 1-month follow-up. The assessment includes the International Trauma Questionnaire (ITQ), Generalized Anxiety Disorder 7-item scale (GAD-7), PHQ-9, Health Questionnaire (EQ-5D-5L), the Oslo Social Support Scale (OSSS-3), and 5-Question Stigma Indicator—Affected People.

In addition to the quantitative assessments, a qualitative component will be included postintervention. Semistructured interviews will be conducted with participants from both the intervention and control groups to explore their experiences and views regarding the TSR intervention and standard care, respectively. This qualitative data will provide deeper insights into the feasibility, acceptability, and effectiveness of the intervention.

The sociodemographic questionnaire will collect essential background information about the participants, including age, gender, marital status, education level, employment status, and duration of leprosy diagnosis. These data are crucial for understanding the context in which participants live and how various demographic factors might influence their mental health outcomes. The sociodemographic information will help in identifying potential correlations between participants' backgrounds and their responses to the intervention, ensuring that any observed effects are appropriately contextualized.

The quantitative data will be analyzed using intention-to-treat principles, with comparisons between the intervention and control groups, whereas the qualitative data will be analyzed using the reflexive thematic analysis [32–35].

##### 2.3.1.1. ITQ-Administered Pre- and Posttreatment

The ITQ is a self-report measure designed to assess symptoms of PTSD and complex PTSD (CPTSD) as defined by the ICD-11 [36]. This questionnaire focuses on core PTSD symptoms such as re-experiencing, avoidance, and a sense of current threat, as well as disturbances in self-organization, including affective dysregulation, negative self-concept, and difficulties in relationships. The tool has a good level or factor structure in Ethiopia [37].

##### 2.3.1.2. GAD-7-Administered Pre- and Posttreatment

The GAD-7 is a widely used self-report tool for assessing the severity of generalized anxiety symptoms. Participants will complete the GAD-7 at baseline and postintervention to evaluate changes in anxiety levels resulting from the TSR intervention. The tool has a good level or factor structure in Ethiopia [38].

##### 2.3.1.3. PHQ-9-Administered Pre -and Posttreatment

The PHQ-9 is a validated tool used to measure the severity of depressive symptoms. This self-report measure will be administered at both baseline and postintervention to assess changes in depression levels among participants. The tool has a good level or factor structure in Ethiopia [39].

##### 2.3.1.4. The EQ-5D-5L Health Questionnaire

The EQ-5D-5L Health Questionnaire is a standardized instrument used to measure health-related quality of life. It includes five dimensions: mobility, self-care, usual activities, pain/discomfort, and anxiety/depression, each with five levels of severity. Participants will complete the EQ-5D-5L at baseline and postintervention to assess changes in their overall health status [40].

##### 2.3.1.5. The OSSS-3

The OSSS-3 is a brief, three-item questionnaire designed to assess the level of social support available to participants. Social support is a critical factor in coping with chronic health conditions and is expected to be significantly impacted by the TSRP intervention. The OSSS-3 will be used at baseline to measure participants' perceived social support.

##### 2.3.1.6. The 5-Question Stigma Indicator

The tool is used to measure the level of stigma experienced by individuals affected by leprosy. This brief questionnaire will be administered to assess the perceived stigma before the TSR intervention.

##### 2.3.1.7. The Theoretical Framework of Acceptability (TFA)

The questionnaire is designed to assess participants' perceptions of the acceptability of the TSR intervention. This measure will be administered postintervention to gather feedback on various aspects of the program, including its content, delivery, and perceived effectiveness [41]. The TFA questionnaire will help the research team understand how well the intervention was received by the participants, which is crucial for refining the program and planning future implementations.

##### 2.3.1.8. Feasibility and Acceptability Indicators

The study will monitor the recruitment rate, including the number of patients referred and the proportion who consented out of all eligible individuals with leprosy referred from recruitment sites or through self-referrals, as well as the attrition rate, which is the number of participants withdrawn compared to those who consented.

The study will evaluate the feasibility of administering the baseline and outcome exploratory assessment battery, focusing on randomization, blinding procedures, and the ability to perform a sample size calculation based on the feasibility data.

The evaluation will assess the number of participants recruited and randomized, session attendance, reported adverse effects, assessment measure acceptability, and the potential for future site. Acceptability will be measured using the TFA scale [41, 42], assessing researchers' and participants' perceptions of the intervention's appropriateness.

##### 2.3.1.9. Postintervention Interviews

Finally, upon completion of the TSR intervention, 20 participants will be invited to participate in one-to-one semistructured qualitative interviews. These interviews will explore participants' experiences with the intervention, including their thoughts on the content, delivery, usefulness, acceptability of the sessions, and the changes in the mental health and life related to their participation in the program. The interviews will also investigate barriers and facilitators to implement, offering insights into both successes and challenges. In addition, participants who withdrew or dropped out of the study will also be invited to share their concerns and perceived barriers to participation. All interviews will be audio-recorded and analyzed using the reflexive thematic analysis, providing a comprehensive understanding of the participants' perceptions that complements the quantitative data. Please see sample questions:

Questions for participants are as follows:

1. In what way did the TSR sessions impact your life?
2. Would you recommend any changes to the TSR sessions?
3. What was the reaction of the people around you to your attending TSR sessions?
4. Have you faced any challenges while taking part in the intervention?
5. Would you recommend TSR to friends or family in the same situation as you?
6. Is there any part of the intervention you are not happy with?
7. What aspect of the intervention/activities did you find most helpful?
8. What were the most important reasons for you to stop (or continue) attending the sessions?
9. In what way did the TSR sessions work (or not work) well for you?

### 2.4. Data and Management

Summary statistics, means, medians, counts, and percentages along with corresponding measures of variability will be reported, along with 95% confidence intervals. The information gathered on the variability of the outcome measures will be sufficient to allow a formal sample size calculation to be made for the subsequent definitive RCT. The proportion of participants recruited and retained in the study will be reported along with 95% confidence intervals. A sample size of 100 will allow the recruitment/retention rates to be estimated with an accuracy of ± 8%.

One-way repeated measures analysis of variance to elucidate the impact of interventions on scores on all outcome tools while accounting for baseline characteristics of participants such as age, gender, and baseline severity of psychological symptoms will be employed. Mean and SD and effect sizes calculated as Cohen's d noted in this trial will inform sample size calculation for future definitive trials. The project will also have a clear Stop-Go criterion:

• If the retention rate is 70% or higher, we will proceed with the full RCT as it is feasible.
• If the retention rate is between 50% and 70%, we will explore methods to increase the retention rate to the required 70%. Alternatively, we may increase the required sample size to account for the higher drop-out rate.
• However, if the recruitment rate is less than 50%, a full trial may not be feasible. In such cases, alternative approaches will be considered.

Subgroup analysis will be carried out with respect to age, duration of illness, number of previous episodes, and gender by adding a treatment with covariate interaction into the primary analysis model.

Data will be initially stored in a secured office location and subsequently digitized for storage on a secure network. In addition, all project-generated data will be assigned unique codes to protect participant confidentiality. Data associated with these codes and any identifiable information will be stored separately.

Descriptive statistics will be conducted for baseline and postintervention measures, including mean scores, standard deviations, and ranges. In addition, recruitment, attendance, and dropout rates will be reported to assess feasibility and acceptability.

The interviews will be audio-recorded and transcribed verbatim. To ensure anonymity, all identifying information will be removed and replaced with generic descriptors. Thematic analysis will be used to analyze the data, following its core principles, meaning data saturation will not be considered [33]. Instead, the consolidated criteria for reporting the qualitative research will be applied to enhance transparency in data analysis [43].

## 3. Discussion

Leprosy not only leads to physical disabilities but also significantly impacts the mental health of affected individuals. The psychosocial consequences of leprosy are profound, with numerous studies highlighting a high prevalence of mental health disorders among those affected [12, 44]. Persons affected by leprosy often experience multiple traumas from the point of diagnosis onwards, contributing to a high prevalence of mental health comorbidities. They also endure stigma, which is not merely a social inconvenience [9, 45, 46]. Furthermore, the mental health burden extends beyond individuals directly affected by leprosy; family members, particularly children of leprosy-affected parents, also experience significant mental health challenges, as they often take on caregiving roles and face societal stigma themselves [3]. Finally, the mental health implications of leprosy are also compounded by socioeconomic factors [47]. Individuals with leprosy frequently have lower educational attainment and income levels, which are associated with poorer mental health outcomes [48–50]. The presence of physical disabilities resulting from leprosy can also lead to chronic pain, which has shown to correlate with increased psychological distress [51]. As noted by many individuals affected by leprosy have become accustomed to societal prejudice, leading to a reluctance to assert their rights and seek help, which further perpetuates their mental health struggles [46].

In Ethiopia, where leprosy remains endemic, despite the efforts there is still a significant gap in addressing the mental health needs of these individuals. This gap is particularly evident in the lack of evidence-based interventions specifically tailored to alleviate the psychological impact of leprosy as mental health care for leprosy patients is generally inconsistent, varying based on available resources and may include counseling and medication. Unfortunately, such services are often limited in rural areas where there is a shortage of skilled professionals and diagnostic expertise.

By offering support that is sensitive to the cultural and social context of the participants, these interventions can help individuals cope more effectively with the challenges posed by the disease, thereby enhancing their quality of life.

The present trial is designed to evaluate the feasibility and acceptability of the TSR for individuals with lived experience of leprosy in Addis Ababa, Ethiopia. The TSR aims to reduce symptoms of PTSD, anxiety, and depression while enhancing social support and overall mental health. This trial will assess whether the TSR is a viable and effective intervention for this population, and whether it can be implemented in a culturally sensitive manner that addresses the unique challenges faced by individuals with leprosy.

This trial has several limitations. First, challenges related to stigma and social isolation might pose difficulties in recruitment and participant engagement, potentially leading to a biased sample. Second, the relatively small sample size may limit the generalizability of the results. Third, the absence of a long-term follow-up may limit the understanding of the sustained effects of the intervention. Despite these limitations, this trial will provide valuable insights into the feasibility of implementing culturally adapted mental health interventions for individuals affected by leprosy. The inclusion of qualitative interviews will further enhance our understanding of the participants' experiences and inform the design of future RCTs aimed at evaluating the effectiveness of the TSR. Most importantly, the strength of the current study is in its potential to ensure there is a sustainable, stigma-friendly response beyond the project as it includes not only professionals but also nonspecialist peer supporters with lived experience in TSR.

In conclusion, this trial represents an important step toward improving mental health care for individuals affected by leprosy. By evaluating the feasibility of the TSR in a culturally sensitive context, this study will contribute valuable knowledge to the field and lay the groundwork for future research on effective mental health interventions for marginalized populations and/or for people affected by NTD. The findings from this trial are expected to provide critical insights into how culturally adapted interventions can be used to support the psychological well-being of individuals living with leprosy, ultimately paving the way for future RCTs.

## Figures and Tables

**Table 1:** Design of the planned intervention.

	Study period			
	Enrollment	Baseline assessment	The intervention	Postintervention assessment
	−*t*1	*t*0	4 sessions	*t*1
Enrollment:				
Eligibility screen	X			
Informed consent	X			
Intervention: TSR				
Assessments:				
Sociodemographic Questionnaire		X		
International Trauma Questionnaire (ITQ)		X		X
Generalized anxiety disorder 7-item (GAD-7)		X		X
Patient Health Questionnaire-9 (PHQ-9)		X		X
Health Questionnaire (EQ-5D-5L)		X		X
Oslo Social Support Scale (OSSS-3)		X		
5-Question stigma indicator—affected people		X		
TFA Acceptability Questionnaire				X
Qualitative interview				X

## Data Availability

The data that support the findings of this study are available on request from the corresponding author. The data are not publicly available due to privacy or ethical restrictions.
