# Corrigendum to “Protocol for an Exploratory RCT of the Traumatic Stress Relief Intervention With Persons With Lived Experience of Leprosy in Addis Ababa, Ethiopia”

**DOI:** 10.1155/jotm/9760139

**Published:** 2025-09-10

S. K. Kaptan, M. Habtamu, S. Getahun, et al., “Protocol for an Exploratory RCT of the Traumatic Stress Relief Intervention With Persons With Lived Experience of Leprosy in Addis Ababa, Ethiopia,” *Journal of Tropical Medicine* 2025 (2025): 1307578, https://doi.org/10.1155/jotm/1307578.

In the article titled “Protocol for an Exploratory RCT of the Traumatic Stress Relief Intervention With Persons With Lived Experience of Leprosy in Addis Ababa, Ethiopia,” the Trial Registration was incorrect. The correct Trial Registration appears below:

“**Trial Registration:** The UK's Clinical Study Registry: ISRCTN86825441”

We apologize for this error.

